# Epiretinal Membrane Vitrectomy With and Without Intraoperative Intravitreal Dexamethasone Implant: A Systematic Review With Meta-Analysis

**DOI:** 10.3389/fphar.2021.635101

**Published:** 2021-04-15

**Authors:** Matteo Fallico, Andrea Maugeri, Giovanni L. Romano, Claudio Bucolo, Antonio Longo, Vincenza Bonfiglio, Andrea Russo, Teresio Avitabile, Martina Barchitta, Antonella Agodi, Francesco Pignatelli, Paola Marolo, Luca Ventre, Guglielmo Parisi, Michele Reibaldi

**Affiliations:** ^1^ Department of Ophthalmology, University of Catania, Catania, Italy; ^2^ Department of Medical and Surgical Sciences and Advanced Technologies “GF Ingrassia”, University of Catania, Catania, Italy; ^3^ Department of Biomedical and Biotechnological Sciences, School of Medicine, University of Catania, Catania, Italy; ^4^ Center for Research in Ocular Pharmacology-CERFO, University of Catania, Catania, Italy; ^5^ Department of Experimental Biomedicine and Clinical Neuroscience, Ophthalmology Section, University of Palermo, Palermo, Italy; ^6^ Eye Unit, SS Annunziata Hospital, Taranto, Italy; ^7^ Department of Surgical Sciences, Eye Clinic Section, University of Turin, Turin, Italy

**Keywords:** Dexamethasone implant, epiretinal membrane (ERM), macular pucker, macular pucker surgery, vitrectomy

## Abstract

**Purpose:** To evaluate the efficacy of vitrectomy combined with intravitreal dexamethasone implant vs. vitrectomy without the implant in patients with epiretinal membrane (ERM) by conducting a systematic review and meta-analysis.

**Methods:** Studies that compared ERM vitrectomy with and without intraoperative dexamethasone implant with a follow-up ≥3 months were included. The primary outcome was mean best corrected visual acuity (BCVA) change between eyes undergoing ERM vitrectomy combined with dexamethasone implant (DEX group) and eyes undergoing ERM vitrectomy alone (control group) at 3 months. Secondary outcomes included mean BCVA change at 6 months and mean optical coherence tomography central macular thickness (CMT) change at both 3-months and 6-months follow-up. Mean differences (MDs) with their 95% confidence interval (95%CI) were calculated. Meta-analyses were based either on random effect model or fixed effect model according to heterogeneity.

**Results:** Four studies were included. At 3 months, ERM vitrectomy combined with dexamethasone implant yielded a greater visual gain compared to vitrectomy alone (MD = 9.7; 95%CI = 2.6–16.8; *p* = 0.01). However, significant heterogeneity was found. A sensitivity analysis excluding the only retrospective non-randomized study confirmed a greater visual gain in the DEX group (MD = 7.1; 95%CI = 2.7–11.6; *p* < 0.01), with no heterogeneity. At 6 months, a non-significant but borderline difference in visual gain was shown between in the two groups (MD = 5.1; 95%CI = −0.3–10.5; *p* = 0.06), with no heterogeneity. Three-month analysis of CMT revealed a greater reduction in the DEX group (MD = −80.2; 95%CI =−149.1–11.2; *p* = 0.02), but with significant heterogeneity. A sensitivity analysis excluding the only retrospective non-randomized study allowed to reduce heterogeneity, but no difference in 3-months CMT change was found between the two groups (MD = −50.0; 95%CI = −106.2–6.2; *p* = 0.08). At 6 months, no difference in CMT change was shown between the two groups (MD = −48.5; 95%CI = −120.5–23.5; *p* = 0.19), with significant heterogeneity.

**Conclusions:** Intraoperative dexamethasone implant in eyes undergoing vitrectomy for ERM provided a better visual outcome at 3 months compared to ERM vitrectomy without the implant, with limited evidence of better anatomic outcome as well. Further studies are needed to ascertain whether dexamethasone implant would ensure a significant long-term visual benefit as a result of a faster reduction of macular thickening.

## Introduction

Idiopathic epiretinal membrane (ERM) represents one of the most common vitreoretinal interface abnormalities affecting the macula, whose incidence increases with age (Meuer et al., 2015; Zapata et al., 2017). The interest toward this condition has peaked following the introduction of high resolution imaging, namely spectral domain-optical coherence tomography (sd-OCT), which improved ERM diagnosis and characterization. In 1997, The Blue Mountains Eye Study based ERM diagnosis on retinal photograph and reported a 11.6% prevalence of ERM in 70–79 years aged people (Mitchell et al., 1997). In 2015, The Baever Dam Eye study found an ERM on sd-OCT in 34% of participants, with a 53% prevalence in ≥85 years aged people (Meuer et al., 2015).

Vitrectomy for idiopathic ERM is indicated when symptoms from retinal surface wrinkling such as reduced visual acuity, with or without metamorphopsia, are present (Smiddy et al., 1990; Massin et al., 2000). The goal of the surgery is ERM removal with long-term favorable outcomes (De Bustros et al., 1988).

A drawback that could jeopardize the visual outcome following ERM vitrectomy is post-vitrectomy macular edema, reported up to 47% of cases (Kim et al., 2009). Given the hypothesis of an inflammatory pathogenesis (Kim et al., 2009; Suzuki et al., 2013), the 0.7 mg intravitreal dexamethasone implant has been used for its treatment, showing positive anatomical and functional results (Furino et al., 2014; Hattenbach et al., 2017; Chatziralli et al., 2019).

Several authors have already described an intraoperative use of the intravitreal dexamethasone implant in patients undergoing vitrectomy for ERM (Guidi et al., 2018; Iovino et al., 2019; Sane et al., 2020; Savastano et al., 2020). However, the outcomes of ERM vitrectomy combined with intravitreal dexamethasone implant appear still controversial (Guidi et al., 2018; Iovino et al., 2019; Sane et al., 2020; Savastano et al., 2020). Even if a greater visual gain featured those patients receiving the implant, almost all reports failed to demonstrated a significant difference between ERM vitrectomy with and without intraoperative dexamethasone implant (Guidi et al., 2018; Sane et al., 2020; Savastano et al., 2020). This fact could be related to the small sample size, which might have limited studies’ evidence.

In this scenario, we carried out a systematic review with meta-analysis of published studies that compared ERM vitrectomy combined with intravitreal dexamethasone implant vs. ERM vitrectomy without the implant, to assess functional and anatomical outcomes.

## Methods

The study was conducted according to the principles reported by the Cochrane Handbook (Higgins et al., 2019) and the Preferred Reporting Items for Systematic Reviews and Meta-analyses (PRISMA) (PRISMA checklist, Supplementary Table S1) (Liberati et al., 2009).

### Search Strategy

A systematic search of studies that compared vitrectomy for ERM with and without intraoperative intravitreal dexamethasone implant was conducted on PubMed, Medline and embase databases, from their inception to November 12th, 2020. Search strategy included the terms ‘epiretinal membrane’, ‘macular pucker’, ‘macular membrane’, ‘cellophane maculopathy’, ‘vitrectomy’, ‘dexamethasone implant’, connected by ‘and/or’ in different combinations. Reference lists of eligible articles were reviewed as well.

### Eligibility Criteria and Outcome Measures

The following inclusion criteria had to be satisfied:

1. Comparing outcomes of vitrectomy with and without intraoperative 0.7 mg dexamethasone implant (Ozurdex^®^, Allergan) in ERM eyes;
2. Presenting a follow-up period ≥3 months;
3. Reporting data on the primary outcome of this meta-analysis.

Only articles published in peer-reviewed journals and in English were considered. Case reports and abstracts were excluded.

The group of eyes treated with ERM vitrectomy combined with intraoperative dexamethasone implant was defined as ‘DEX group’, while the group of eyes treated with ERM vitrectomy alone was defined as ‘control group’. The primary outcome was to compare mean best corrected visual acuity (BCVA) change between the DEX group and the control group at 3-months follow-up. Secondary outcomes included mean BCVA change at 6 months and mean OCT central macular thickness (CMT) change at both 3-months and 6-months follow-up. Best corrected visual acuity was reported as Early Treatment Diabetic Retinopathy Study (ETDRS) letters.

### Data Extraction and Quality Assessment

Two investigators (AR, AL) separately analyzed titles and abstracts of eligible studies, carrying out full-text assessment when inclusion criteria were satisfied. Data extraction from each selected study was carried out by the same two investigators. In case of disagreement, a third investigator (MF) was consulted to reach a shared consensus. In case further information and clarification were needed for either eligibility assessment or data extraction, the authors of the study were contacted. The following data were extracted: location and publication year, first author, study design, number of patients, follow-up duration, mean age and gender; for each cohort the following data were collected: mean BCVA change, mean CMT change, number of intravitreal dexamethasone implant injections (DEX group), peeling technique, cataract surgery, mean intraocular pressure (IOP) change, rate of IOP rise, recorded complications.

Quality assessment of included studies was based on the Cochrane Collaboration Reviewers' Handbook for Systematic Reviews (Higgins et al., 2019) and on the New-castle Ottawa Scale (NOS) (Stang, 2010) as to whether they had or not a randomized design, respectively. A NOS score ≥6 was considered as low-to-moderate risk of bias (Fallico et al., 2018).

### Statistical Analysis

Both BCVA gain and CMT change were assessed as mean differences (MDs) between baseline and 3-months follow-up values, with their 95% Confidence Interval (95%CI). We also analyzed the same outcomes after 6-months follow-up if reported. Q-statistics and *I* ^2^ index were applied to test and to measure heterogeneity across studies. If significant heterogeneity was evident (*p*-value for Q-statistics <0.01 and *I* ^2^ > 50%), the DerSimonian-Laird random effect model was applied instead of the Mantel–Haenszel fixed effect model. We also carried out a sensitivity analysis by excluding the only one study which featured a non-randomized retrospective design. The extent of publication bias was explored by funnel plots and tested using the Egger’s test. All the statistical analyses were performed using STATA (version 16) with significance level *α* < 0.05 if not otherwise indicated.

## Results

**FIGURE 1:**
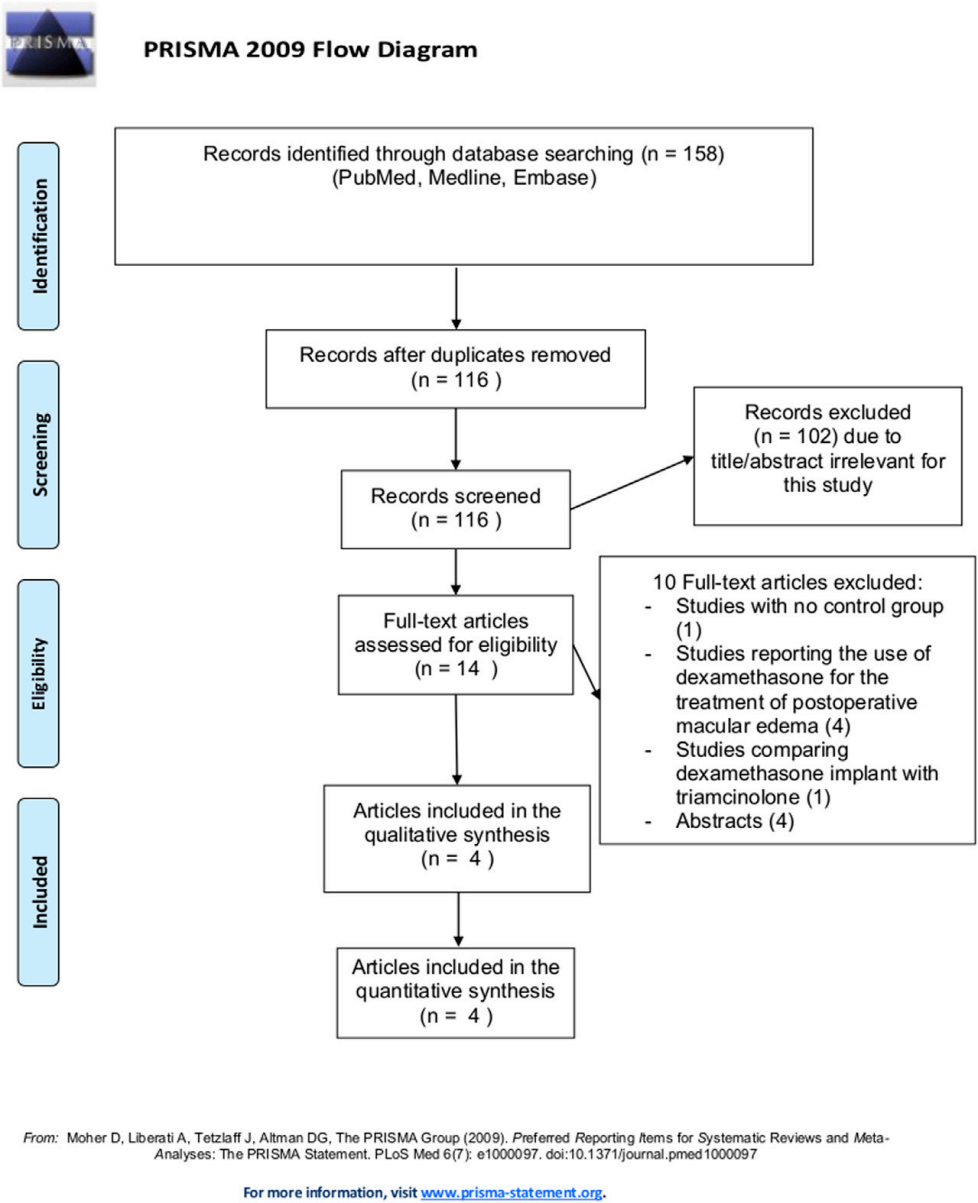
Flow chart of study selection process.

The whole study selection flow chart is illustrated in Figure 1. By our tools, we identified 158 articles, and eliminated 42 that were duplicates. The remaining 116 articles were further filtered, and a total of 14 potential eligible articles were selected and full-text reviewed. Of these, 4 articles fully satisfied eligibility criteria and were included in the present meta-analysis.

### Characteristics of Included Studies

Four studies were included, with a total of 85 eyes in each group. Two studies presented a randomized design (Guidi et al., 2018; Iovino et al., 2019); of the two non-randomized studies, one was prospective (Sane et al., 2020) and one was retrospective (Savastano et al., 2020). All studies except one had a follow-up of 6 months (Guidi et al., 2018; Iovino et al., 2019; Sane et al., 2020); the report from Savastano and co-workers featured a 3-months follow-up (Savastano et al., 2020). All studies based ERM diagnosis on sd-OCT. A CMT greater than 250 and 300 µm was considered for eligibility by Guidi et al. (2018) and Sane et al. (2020), respectively. Savastano and co-workers included eyes with ≤400 µm CMT and absence of edema (Savastano et al., 2020). Iovino and co-workers deemed eligible eyes with advanced ERM stage (III and IV according to Govetto and co-workers’ classification) (Govetto et al., 2017) and presence of intraretinal cysts (Iovino et al., 2019). All studies included only idiopathic ERM, excluding the secondary ones (Guidi et al., 2018; Iovino et al., 2019; Sane et al., 2020; Savastano et al., 2020). Diabetic retinopathy and retinal vein occlusion were considered as exclusion criteria in all included studies. Eyes that had undergone previous vitreoretinal surgery were also excluded. Diagnosis of glaucoma was an exclusion criterion common in all studies (Guidi et al., 2018; Iovino et al., 2019; Sane et al., 2020; Savastano et al., 2020). In particular, Guidi and coworkers excluded eyes with other ocular comorbidities (Guidi et al., 2018); Iovino and coworkers ruled out eyes with causes of visual impairment other than ERM (Iovino et al., 2019); Sane and coworkers and Savastano and coworkers excluded eyes with age related macular degeneration (Sane et al., 2020; Savastano et al., 2020).

Two out of 4 studies enrolled only pseudophakic patients (Guidi et al., 2018; Iovino et al., 2019); Savastano and co-workers included only phakic patients, performing a combined phaco-vitrectomy in all cases (Savastano et al., 2020); Sane and co-workers enrolled 13 and 6 phakic eyes in the DEX group and control group, respectively, of which 6 and 2, respectively, underwent a combined phaco-vitrectomy (Sane et al., 2020). All included studies adopted 25-gauge vitrectomy system. Surgical technique involved ERM peeling in all cases and internal limiting membrane (ILM) peeling in 3 out of 4 studies (Guidi et al., 2018; Iovino et al., 2019; Savastano et al., 2020). Iovino and coworkers and Savastano and coworkers used the membrane blue-dual dye (Iovino et al., 2019; Savastano et al., 2020), while Guidi and coworkers used the brilliant blue G dye (Guidi et al., 2018). Sane and coworkers described a ERM peeling, with no mention about ILM peeling or about the dye (Sane et al., 2020). Guidi and coworkers, iovino and coworkers and Sane and coworkers reported the use of forceps (Guidi et al., 2018; Iovino et al., 2019; Sane et al., 2020), while Savastano and coworkers did not provide this information (Savastano et al., 2020). No study reported the use of the Tano scraper for peeling procedure. No information was provided on duration of light exposure during the surgery.

In all included studies, eyes in DEX group received a single 0.7 mg dexamethasone implant in combination with ERM vitrectomy and no additional DEX implant was given throughout the follow-up (Guidi et al., 2018; Iovino et al., 2019; Sane et al., 2020; Savastano et al., 2020). No change in mean IOP throughout the follow-up was reported by Guidi and co-workers (Guidi et al., 2018). Iovino and co-workers recorded a transient IOP rise in the DEX group at one month, which leveled off at 3 and 6 months (mean IOP, 18.0 ± 1.5 mmHg at 1 month, 16.0 ± 2.6 mmHg and 15.4 ± 2.4 mmHg at 3 and 6 months, respectively) (Iovino et al., 2019). Savastano and co-workers and Sane and co-workers reported IOP-lowering drop use in 1 out of 15 and 3 out of 20 eyes, respectively (Sane et al., 2020; Savastano et al., 2020). With regards to adverse reaction events, Sane and co-workers reported one case of retinal detachment and one case of pale disc, both in the DEX group (Sane et al., 2020).

### Quality Assessment

Both non-randomized studies were given a NOS score of 9, featuring a low-to-moderate risk of bias (Sane et al., 2020; Savastano et al., 2020). Four points were assigned for selection, one for comparability and 4 for outcome assessment. The two randomized trials (Guidi et al., 2018; Iovino et al., 2019) were judged as low risk and unclear risk for random sequence generation and allocation concealment, respectively. Performance bias and detection bias were considered as high risk in the study of Iovino et al. (2019), and unclear in the study of Guidi et al. (2018), while attrition bias and reporting bias were deemed as low risk in both trials. Risk of other bias was low as well. With respect to publication bias, no concern was raised for the BCVA outcome, given the nearly symmetrical shape of the funnel plot (Supplementary Figure S1A) and Egger’s test value (*p* = 0.338). Instead, presence of publication bias cannot be completely excluded for the CMT outcome, as indicated by funnel plot inspection (Supplementary Figure S1B) and Egger’s test (*p* = 0.044).

### Best Corrected Visual Acuity

**FIGURE 2:**
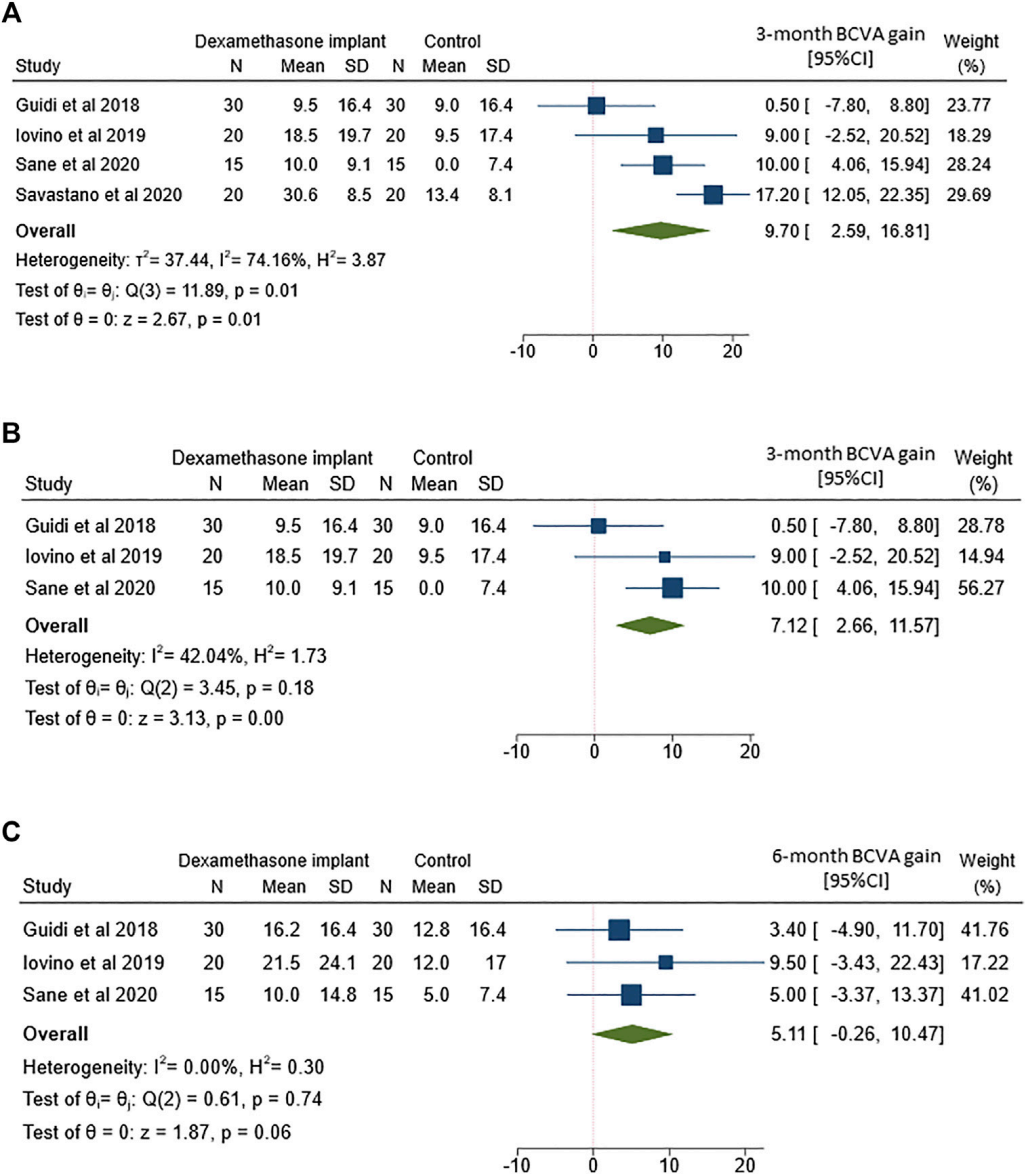
Forest plots of the comparison of mean best corrected visual acuity (BCVA) gain between vitrectomy with and without intravitreal dexamethasone implant **(A)** BCVA gain assessed at 3-months follow-up **(B)** Sensitivity analysis of BCVA gain at 3-months follow-up by excluding the study by Savastano and coworkers **(C)** BCVA gain assessed at 6-months follow-up.

The analysis of 3-months mean BCVA change between ERM vitrectomy with and without intravitreal dexamethasone implant was based on data from all 4 included studies (Figure 2A). Although both treatments yielded to better BCVA, the improvement was more marked after ERM vitrectomy with dexamethasone implant than vitrectomy alone (MD = 9.7; 95%CI = 2.6–16.8; *p* = 0.01). However, Q-statistics and *I* ^2^ indicated significant heterogeneity across studies (*p* = 0.01 for Q-statistics and *I* ^2^ = 74.2%).

To partially reduce heterogeneity, we removed the only study that featured a non-randomized retrospective design (Figure 2B). While heterogeneity decreased after removing Savastano and co-workers (Savastano et al., 2020), the better outcome observed in the dexamethasone implant group remained statistically significant (MD = 7.1; 95%CI = 2.7–11.6; *p* < 0.01).

The analysis of 6-months mean BCVA change between the two groups included 3 out of 4 studies. The comparison showed a non-significant but borderline difference between the two treatments (Figure 2C), with an improvement in eyes which received intravitreal dexamethasone implant (MD = 5.1; 95%CI = −0.3–10.5; *p* = 0.06). It is worthy of note that no heterogeneity was evident across studies reporting BCVA change after 6-months follow-up (*p* = 0.74 for Q-statistics and *I* ^2^ = 0%).

### Central Macular Thickness

**FIGURE 3:**
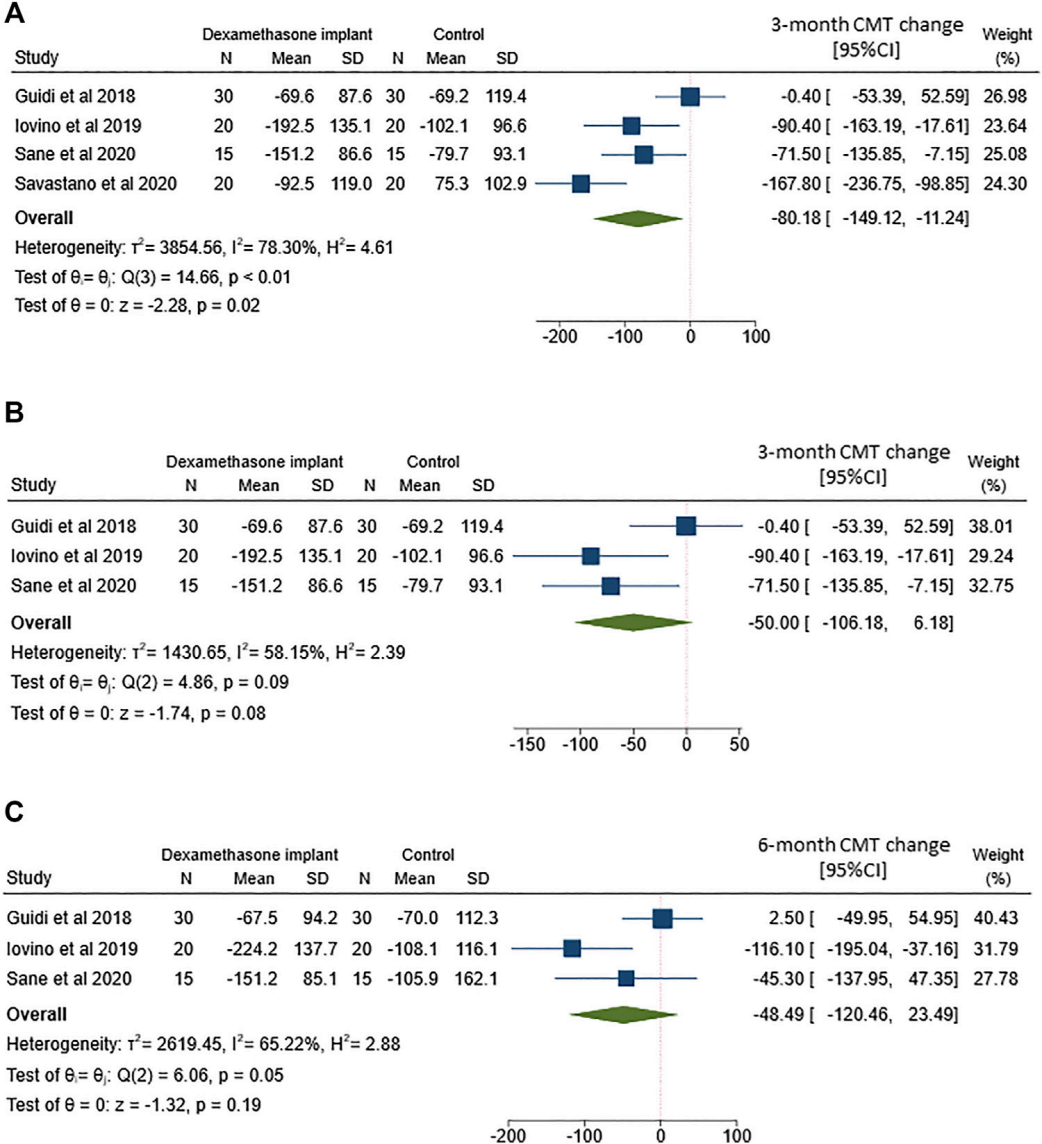
Forest plots of the comparison of mean central macular thickness (CMT) change between vitrectomy with and without intravitreal dexamethasone implant **(A)** CMT change assessed at 3-months follow-up **(B)** Sensitivity analysis of CMT change at 3-months follow-up by excluding the study by Savastano and coworkers **(C)** CMT change assessed at 6-months follow-up.

The analysis of 3-months mean CMT change between ERM vitrectomy with and without intravitreal dexamethasone implant was based on data from all 4 included studies (Figure 3A). Although both treatments tended to reduce CMT, the improvement was more marked after ERM vitrectomy with dexamethasone implant than vitrectomy alone (MD = −80.2; 95%CI = −149.1–-11.2; *p* = 0.02). However, Q-statistics and *I* ^2^ indicated significant heterogeneity across studies (*p* < 0.01 for Q-statistics and *I* ^2^ = 78.3%).

Here too, we removed the study by Savastano et al. (2020) to partially reduce heterogeneity across studies (Figure 3B). Heterogeneity improved (*p* = 0.09 for Q-statistics and *I* ^2^ = 58.2%), but no significant difference in CMT change was evident between ERM vitrectomy alone or in combination with intravitreal dexamethasone implant (MD = −50.0; 95%CI = −106.2–6.2; *p* = 0.08).

We also compared CMT change after 6-months follow-up (Figure 3C), including 3 out of 4 studies. This analysis yielded to non-significant differences between the two treatments (MD = −48.5; 95%CI = −120.5–23.5; *p* = 0.19) and high heterogeneity across the studies (*p* = 0.05).

## Discussion

This systematic review and meta-analysis demonstrated that eyes undergoing ERM vitrectomy combined with intraoperative dexamethasone had a better visual outcome at 3-months follow-up compared with those undergoing ERM vitrectomy without dexamethasone implant.

The 0.7 mg intravitreal dexamethasone implant is approved in Europe for the treatment of diabetic macular edema, macular edema secondary to retinal vein occlusion and posterior segment inflammation due to non-infectious uveitis (Bonfiglio et al., 2017; Bucolo et al., 2018). The drug slow-release represents one of the major advantage of the implant, along with the potent anti-inflammatory activity of the active ingredient and the good safety profile (Bonfiglio et al., 2017; Bucolo et al., 2018). The use of this implant has been extended to other conditions, including pseudophakic cystoid macular edema (Bellocq et al., 2017) and inflammation secondary to retinal detachment surgery (Bonfiglio et al., 2015). The efficacy of dexamethasone implant has been also demonstrated in vitrectomized eyes, where anti-vascular endothelial growth factor agents are less effective because of the faster pharmacokinetics profile (Boyer et al., 2011; Platania et al., 2015; Bonfiglio et al., 2017). In particular, intravitreal dexamethasone implant provided favorable outcomes when administered for the treatment of macular edema secondary to vitrectomy for ERM (Furino et al., 2014; Hattenbach et al., 2017; Chatziralli et al., 2019).

Postoperative macular edema secondary to ERM surgery has been reported in a relevant percentage of cases, ranging from 13 to 64% of cases (Kim et al., 2009; Frisina et al., 2014). The wrinkling of retinal surface seems to play a central role in the pathogenesis of postoperative macular edema (Konstantinidis et al., 2009). The mechanical distortion has been supposed to trigger an inflammatory process eliciting several cytokines and growth factors release, with a subsequent retinal edema (Konstantinidis et al., 2009). The surgical insult might also contribute to this inflammatory process. In this context, the rationale for using corticosteroids is to inhibit the inflammatory pathways and to promote a faster resolution of macular thickening (Guidi et al., 2018; Iovino et al., 2019). Of note, a greater improvement in macular thickness in the immediate postoperative period correlates with a better visual recovery (Kim et al., 2009).

However, when it comes to the use of intravitreal dexamethasone implant in combination with vitrectomy for ERM, there is no univocal agreement on its benefits (Guidi et al., 2018; Iovino et al., 2019; Sane et al., 2020; Savastano et al., 2020). Guidi et al. (2018), Savastano et al. (2020) and Sane et al. (2020) reported no significant difference between ERM vitrectomy with and without intraoperative dexamethasone. Nonetheless, all of these reports showed a trend of better visual outcome in those receiving the implant. On the other hand, iovino and coworkers (Iovino et al., 2019) found a greater visual gain in eyes undergoing ERM vitrectomy combined with dexamethasone implant.

The findings of the present meta-analysis provide a clearer picture of this issue. The 3-months analysis revealed a better visual gain in the DEX group. Indeed, we first conducted a meta-analysis including all 4 studies, which showed a better visual outcome but with significant heterogeneity. Then, we decided to perform a further analysis excluding the report of Savastano et al. (2020), which was likely to affect heterogeneity across studies given its retrospective non-randomized design. Yet, the sensitivity analysis confirmed a better visual gain at 3 months in eyes receiving the implant compared to eyes without implant, with no significant heterogeneity.

Our 6-months analysis revealed a trend of better visual gain in the DEX group, with a borderline non-significance (*p* = 0.06). This finding was influenced by no heterogeneity. A possible reason for failing to achieve a statistical significance could be the fact that fewer eyes were included in this analysis because the report of Savastano and coworkers (Savastano et al., 2020) was excluded given the shorter follow-up. Additionally, this could be also explained by a loss of effectiveness of the implant before 6 months. The effectiveness of the slow-release implant tends to last from 4 to 5.9 months (Bonfiglio et al., 2017), with a peak of dexamethasone concentration at 2 months (Kiddee et al., 2013). This was also the reason why we chose to set the primary outcome of this study at 3 months.

With regards to macular thickness, our findings showed a significant CMT reduction in the DEX group at 3 months, which was not maintained at 6 months. However, the strength of the 3-months result was limited by significant heterogeneity and failed to be confirmed by the sensitivity analysis excluding Savastano et al. (2020). Nonetheless, the 3-months sensitivity analysis revealed a trend of CMT reduction in the DEX group with a borderline statistical non-significance (*p* = 0.08). The 6-months result could be related to an earlier loss of efficacy.

Importantly, the studies included in this meta-analysis could differ in eligibility criteria and clinical variables. In particular, the report of Savastano et al. (2020) was the only one with a retrospective design and the only one which performed a combined phaco-vitrectomy in all cases. This is a relevant shortcoming because phacoemulsification could further increase the risk of postoperative macular edema and it is hard to discern whether a possible thickening was ERM-related or cataract surgery-related. However, intravitreal dexamethasone implant combined with cataract surgery has been shown to prevent a worsening of diabetic macular edema (Fallico et al., 2020a). Similarly, it could be useful to for preventing a thickening secondary to both ERM and cataract surgery-related inflammation. Moreover, to support the strength of our findings, we carried out sensitivity analyses excluding Savastano’s study (Savastano et al., 2020). This allowed to reduce heterogeneity and to improve the quality of the evidence for the primary outcome of the study. On the other hand, however, we cannot completely exclude the presence of publication bias, especially for the CMT outcome. Concerning the lens status, two included studies enrolled only pseudophakic eyes (Guidi et al., 2018; Iovino et al., 2019), while Sane and coworkers included both phakic and pseudophakic eyes and performed a combined phaco-vitrectomy in 8 out of 19 phakic eyes (Sane et al., 2020). An analysis which would have allowed to test the baseline phakic status and the combined phaco-vitrectomy surgery as confounding factors couldn’t be conducted because only 2 out of 4 included studies reported data on pseudophakic eyes. This represents a limitation of the present meta-analysis. However, included studies shared other common eligibility criteria, such as including only idiopathic ERM and excluding eyes with diabetic retinopathy, retinal vein occlusion, glaucoma, age related macular degeneration, and history of previous vitreoretinal surgery. These common eligibility criteria could help to reduce the selection bias, even if a possible bias related to the baseline lens status and combined phaco-vitrectomy couldn’t have been excluded.

With regards to surgical aspects, all studies adopted a 25-gauge vitrectomy system. Peeling procedures were comparable in 3 out of 4 included studies, which reported a blue dye-assisted ERM and ILM peeling (Guidi et al., 2018; Iovino et al., 2019; Savastano et al., 2020). Only Sane and coworkers described only the ERM peeling, with no mention about the use of any dye (Sane et al., 2020). Furthermore, no information on duration of light exposure was available amongst included studies. Differences in surgical procedure could potentially be a source of bias.

Unfortunately, no analysis on postoperative IOP rise could have been performed, due to low number of studies reporting the rate of IOP rise. Neither any analysis on mean change of IOP was conducted due to lack of data. However, this was not the primary outcome of this study and the safety profile of this implant has been already sufficiently studied, showing a good safety profile (Bonfiglio et al., 2017).

A further limitation of the present study was the reduced number of included studies, with a relatively small sample size. However, these were all comparative studies, which makes our results more valuable thanks to the presence of a control group. Furthermore, results of meta-analyses are more accurate than those of individual reports (Fallico et al., 2020b).

In conclusion, the intraoperative use of intravitreal dexamethasone implant in patients undergoing vitrectomy for ERM provided a better visual outcome at 3 months. A limited evidence of a better anatomic outcome was also shown. At 6-months follow-up, a borderline difference in visual gain, even though non-significant, was demonstrated, while macular thickness change was comparable with those non-receiving the implant. Despite promising results, further carefully designed studies are needed to ascertain whether dexamethasone implant would ensure a significant long-term visual benefit as a result of a faster reduction of macular thickening.

## Data Availability

The original contributions presented in the study are included in the article/Supplementary Material, further inquiries can be directed to the corresponding author.

## References

[B1] Bellocq D. Pierre-Kahn V. Matonti F. Burillon C. Voirin N. Dot C. (2017). Effectiveness and safety of dexamethasone implants for postsurgical macular oedema including Irvine-Gass syndrome: the EPISODIC-2 study. Br. J. Ophthalmol. 101, 135–260. 10.1136/bjophthalmol-2016-308544 27190126

[B2] Bonfiglio V. Fallico M. R. Russo A. De Grande V. Longo A. Uva M. G. (2015). Intravitreal dexamethasone implant for cystoid macular edema and inflammation after scleral buckling. Eur. J. Ophthalmol. 25, e98–e100. 10.5301/ejo.5000599 25790811

[B3] Bonfiglio V. Reibaldi M. Fallico M. Russo A. Pizzo A. Fichera S. (2017). Widening use of dexamethasone implant for the treatment of macular edema. Dddt Vol. 11, 2359–2372. 10.2147/DDDT.S138922 PMC5566324 28860707

[B4] Boyer D. S. Faber D. Gupta S. Patel S. S. Tabandeh H. Li X.-Y. (2011). Dexamethasone intravitreal implant for treatment of diabetic macular edema in vitrectomized patients. Retina 31, 915–923. 10.1097/IAE.0b013e318206d18c 21487341

[B5] Bucolo C. Gozzo L. Longo L. Mansueto S. Vitale D. C. Drago F. (2018). Long-term efficacy and safety profile of multiple injections of intravitreal dexamethasone implant to manage diabetic macular edema: a systematic review of real-world studies. J. Pharmacol. Sci. 138, 219–232. 10.1016/j.jphs.2018.11.001 30503676

[B6] Chatziralli I. Dimitriou E. Theodossiadis G. Chatzirallis A. Kazantzis D. Theodossiadis P. (2019). Treatment of macular edema after pars plana vitrectomy for idiopathic epiretinal membrane using intravitreal dexamethasone implant: long-term outcomes. Ophthalmologica 242, 16–21. 10.1159/000496705 30889589

[B7] De Bustros S. Thompson J. T. Michels R. G. Rice T. A. Glaser B. M. (1988). Vitrectomy for idiopathic epiretinal membranes causing macular pucker. Br. J. Ophthalmol. 72, 692–695. 10.1136/bjo.72.9.692 3179258 PMC1041558

[B8] Fallico M. Avitabile T. Castellino N. Longo A. Russo A. Bonfiglio V. (2020a). Intravitreal dexamethasone implant one month before versus concomitant with cataract surgery in patients with diabetic macular oedema: the dexcat study. Acta Ophthalmol. 99, 145–156. 10.1111/aos.14516 32588978

[B9] Fallico M. Lotery A. J. Longo A. Avitabile T. Bonfiglio V. Russo A. (2020b). Risk of acute stroke in patients with retinal artery occlusion: a systematic review and meta-analysis. Eye 34, 683–689. 10.1038/s41433-019-0576-y 31527762 PMC7093449

[B10] Fallico M. Russo A. Longo A. Pulvirenti A. Avitabile T. Bonfiglio V. (2018). Internal limiting membrane peeling versus no peeling during primary vitrectomy for rhegmatogenous retinal detachment: a systematic review and meta-analysis. PLoS One 13, e0201010. 10.1371/journal.pone.0201010 30024983 PMC6053210

[B11] Frisina R. Pinackatt S. J. Sartore M. Monfardini A. Baldi A. Cesana B. M. (2014). Cystoid macular edema after pars plana vitrectomy for idiopathic epiretinal membrane. Graefes Arch. Clin. Exp. Ophthalmol. 253, 47–56. 10.1007/s00417-014-2655-x 24859385

[B12] Furino C. Boscia F. Recchimurzo N. Sborgia C. Alessio G. (2014). Intravitreal dexamethasone implant for refractory macular edema secondary to vitrectomy for macular pucker. Retina 34, 1612–1616. 10.1097/IAE.0000000000000105 24752008

[B13] Govetto A. Lalane R. A. Sarraf D. Figueroa M. S. Hubschman J. P. (2017). Insights into epiretinal membranes: presence of ectopic inner foveal layers and a New optical coherence tomography staging scheme. Am. J. Ophthalmol. 175, 99–113. 10.1016/j.ajo.2016.12.006 27993592

[B14] Guidi G. Casini G. Ripandelli G. Piaggi P. Dalle Lucche F. Sartini M. (2018). Residual intraretinal edema after 25-gauge vitrectomy and macular pucker removal. Retina 38, 993–999. 10.1097/IAE.0000000000001627 28376039

[B15] Hattenbach L.-O. Springer-Wanner C. Hoerauf H. Callizo J. Jungmann S. Brauns T. (2017). Intravitreal sustained-release steroid implants for the treatment of macular edema following surgical removal of epiretinal membranes. Ophthalmologica 237, 232–237. 10.1159/000464259 28463851

[B16] Higgins J. P. T. Thomas J. Chandler J. Cumpston M. Li T. Page M. J. (Editors) (2019). “Cochrane Handbook for systematic reviews of interventions,” in Cochrane Handb. Syst. Rev. Interv. 10.1002/14651858.ED000142 PMC10284251 31643080

[B17] Iovino C. Giannaccare G. Pellegrini M. Bernabei F. Braghiroli M. Caporossi T. (2019). Efficacy and safety of combined vitrectomy with intravitreal dexamethasone implant for advanced stage epiretinal membrane. Dddt Vol. 13, 4107–4114. 10.2147/DDDT.S229031 PMC6899066 31819377

[B18] Kiddee W. Trope G. E. Sheng L. Beltran-Agullo L. Smith M. Strungaru M. H. (2013). Intraocular pressure monitoring post intravitreal steroids: a systematic review. Surv. Ophthalmol. 58, 291–310. 10.1016/j.survophthal.2012.08.003 23768920

[B19] Kim S. J. Martin D. F. Hubbard G. B. Srivastava S. K. Yan J. Bergstrom C. S. (2009). Incidence of postvitrectomy macular edema using optical coherence tomography. Ophthalmology 116, 1531–1537. 10.1016/j.ophtha.2009.02.008 19501405

[B20] Konstantinidis L. Berguiga M. Beknazar E. Wolfensberger T. J. (2009). Anatomic and functional outcome after 23-gauge vitrectomy, peeling, and intravitreal triamcinolone for idiopathic macular epiretinal membrane. Retina 29, 1119–1127. 10.1097/IAE.0b013e3181ac23da 19734764

[B21] Liberati A. Altman D. G. Tetzlaff J. Mulrow C. Gotzsche P. C. Ioannidis J. P. A. (2009). The PRISMA statement for reporting systematic reviews and meta-analyses of studies that evaluate healthcare interventions: explanation and elaboration. BMJ 339, b2700. 10.1136/bmj.b2700 19622552 PMC2714672

[B22] Massin P. Allouch C. Haouchine B. Metge F. Paques M. Tangui L. (2000). Optical coherence tomography of idiopathic macular epiretinal membranes before and after surgery. Am. J. Ophthalmol. 130, 732–739. 10.1016/S0002-9394(00)00574-2 11124291

[B23] Meuer S. M. Myers C. E. Klein B. E. K. Swift M. K. Huang Y. Gangaputra S. (2015). The epidemiology of vitreoretinal interface abnormalities as detected by spectral-domain optical coherence tomography. Ophthalmology 122, 787–795. 10.1016/j.ophtha.2014.10.014 25556116 PMC4372472

[B24] Mitchell P. Smith W. Chey T. Wang J. J. Chang A. (1997). Prevalence and associations of epiretinal membranes. Ophthalmology 104, 1033–1040. 10.1016/S0161-6420(97)30190-0 9186446

[B25] Platania C. B. M. Di Paola L. G. M. Leggio G. L. Drago F. Salomone S. (2015). Molecular features of interaction between VEGFA and anti-angiogenic drugs used in retinal diseases: a computational approach. Front. Pharmacol. 6. 10.3389/fphar.2015.00248 PMC4624855 26578958

[B26] Sane S. S. Ali M. H. Kuppermann B. D. Narayanan R. (2020). Comparative study of pars plana vitrectomy with or without intravitreal dexamethasone implant for idiopathic epiretinal membrane. Indian J. Ophthalmol. 68, 1103–1107. 10.4103/ijo.IJO_1045_19 32461441 PMC7508104

[B27] Savastano A. Bitossi A. Giansanti F. Vannozzi L. Caporossi T. Barca F. (2020). Evaluation of intraoperative slow-release dexamethasone implant combined with idiopathic epiretinal membrane removal. Graefes Arch. Clin. Exp. Ophthalmol. 259, 379. 10.1007/s00417-020-04911-5 32892264

[B28] Smiddy W. E. Michels R. G. Green W. R. (1990). Morphology, pathology, and surgery of idiopathic vitreoretinal macular disorders. Retina 10, 288–296. 10.1097/00006982-199010000-00012 2089546

[B29] Stang A. (2010). Critical evaluation of the Newcastle-Ottawa scale for the assessment of the quality of nonrandomized studies in meta-analyses. Eur. J. Epidemiol. 25, 603–605. 10.1007/s10654-010-9491-z 20652370

[B30] Suzuki T. Hayakawa K. Onouchi Y. Ogata H. Nakagawa M. Kawai K. (2013). Topical dorzolamide for macular edema in the early phase after vitrectomy and epiretinal membrane removal. Opth 7, 549–553. 10.2147/OPTH.S42188 PMC3633550 23620653

[B31] Zapata M. A. Figueroa M. S. Esteban González E. Huguet C. Giralt J. Gallego Pinazo R. (2017). Prevalence of vitreoretinal interface abnormalities on spectral-domain OCT in healthy participants over 45 Years of age. Ophthalmol. Retina 1, 249–254. 10.1016/j.oret.2016.11.001 31047428

